# A Lenz into the predictive power of language teacher emotion regulation and self-evaluation on L2 grit, teaching style preferences, and work engagement: a case of Chinese EFL instructors

**DOI:** 10.1186/s40359-023-01356-3

**Published:** 2023-10-11

**Authors:** Yue Ma

**Affiliations:** School of Foreign Languages, Huizhou University, Huizhou, Guangdong 516007 China

**Keywords:** Language teacher emotion regulation, Self-evaluation, L2 grit, Teaching style preferences, Work Engagement, EFL Teachers

## Abstract

An individual’s capacity to successfully control their emotional experiences and react to them requires them to engage in a number of processes, including those that are physiological, behavioral, and cognitive. When educators engage in self-evaluation, they investigate and assess the quality of their professional work. These two teacher-related conceptions have the potential to open up valuable perspectives in the course of the professional pursuits of teachers. Even though earlier research has shown their significance, the potential implications of these factors on the resiliency and teaching style preferences of language instructors have not been emphasized. As a result, the purpose of this study was to determine the extent to which a language teacher’s ability to regulate their emotions while carrying out self-evaluation procedures may accurately predict their level of resilience as well as their preferred method of instruction. To accomplish this, 399 English as a foreign language (EFL) teachers were asked to reflect on their experiences by responding to the following related questionnaires: The Language Teacher Emotion Regulation Inventory (LTERI), The Core of Self-evaluation Questionnaire (CSEQ), the L2-teacher Grit Scale (L2TGS), Grasha Teaching Style Inventory (TSI) and the Engaged Teacher Scale (ETS). The results demonstrated that those EFL teachers who maintained healthy emotional control were grittier and more engaged. They also tended to teach in a manner focused on the students. The pedagogical implications of this research are discussed further in depth.

## Overview

In light of the undeniable connection between the efficacy of a teacher and how they feel and think about their work, educators must use efficient techniques to control the feelings they experience in the classroom and improve their cognitive abilities. In the realm of successful instruction, especially particular language instruction, it appears logical to infer that a greater understanding of emotional skills assists instructors in managing and modifying their academic achievement. This is especially true concerning the instruction of second language learners. According to [1], emotional competencies impact the efficiency of instructors and students’ cognitive and emotional growth, which leads to profitable and efficient teaching.

Emotion regulation (ER) may be seen as a complicated process that refers to the many methods utilized for starting, hindering, or adjusting persons’ positions or behaviors in response to a particular event [2]. In the words of [3], “their competence to regulate emotional experiences and manifestations” is what is meant by teacher emotion regulation (TER). More precisely, TER serves as a fortress that inoculates educators against the stresses they confront in today’s dynamic and difficult classrooms. Since teaching a language is emotionally draining, TER plays an even more critical role in the field [4]. It has been suggested that ER processes be seen as a continuum ranging from mindful, purposeful monitoring to unnoticed, effortless, automated modulation [5]. According to [6], the ER may be adjusted regarding delay, growing time, dimensions, and duration. Furthermore, ER may modify the extent to which the many components of the emotional response align as the emotion unfolds [7].

The information that was previously accessible provided proof of the advantages of ER in the realm of education. For instance, classroom misconduct was the focus of research by [1]. Results showed that educators who engaged in reappraisal felt less emotional distress and less emotional repression due to student misconduct [5] followed a similar line of inquiry and concluded that the most effective method for EFL teachers to deal with dissatisfaction was using emotion-regulating tactics. A mixed-method research studied learners’ perspectives on instructors’ emotions and ER in the classroom [8]. Their analysis indicated that controlling one’s emotions by focusing on their causes rather than consequences was more effective. They also found that reappraisals by instructors led to more upbeat behavior. Teacher reflection, self-efficacy, burnout, and ER were all factors that [9] looked at. Their research indicated that ER might be predicted by a teacher’s level of self-efficacy and reflectiveness. This research also validated the known link between ER and burnout. In a related vein, researchers have determined the effects of teacher self-efficacy and ER on students’ mental health in an English as a foreign language setting [10].

Self-evaluation (S-E), a kind of assessment in which educators play a prominent role, requires participants to actively engage in “assessing or evaluating” themselves or their actions, attitudes, or outcomes. [11] argue that S-E’s core tenets are cognition tracking, mental processing, inspection, and autonomous teaching. In addition, S-E is seen as a higher-order characteristic that includes elements such as self-worth, generalized self-efficacy, neuroticism, and locus of control [12]. The development of S-E might come from both internal and external sources. The extrinsic stage of S-E is characterized by consideration of external values and job promotion. The intrinsic stage of S-E is characterized by the use of intrinsic values and the establishment of objectives.

EFL teachers with high self-efficacy (S-E) have enhanced capabilities in effectively addressing a wide range of obstacles and exhibiting thoughtful decision-making skills [13]. There is a positive correlation between the capacity to sustain a heightened state of self-esteem and improved emotional management and professional success [14]. [15] claims that teaching students to self-regulate their emotions using SE is effective. This suggests that teachers’ and students’ S-E might positively impact students’ emotional and intellectual development. It was also determined that S-E is influenced by self-efficacy beliefs, intellectual capacity, and analytical skills [16].

Another component examined in this study is the concept of L2 teacher grit (L2T G). Teachers with grit in the second/foreign language classroom combine a love of teaching with a commitment to seeing students succeed in the long run [17]. Research on L2TG is still in its infancy, and its links with other teacher-related constructs remain murky. [18] proposed the grit hypothesis, emphasizing the symbiotic connection between passion and tenacity in determining an individual’s success. To be enthusiastic about something is to develop a strong emotional attachment. Individuals are motivated to engage in tasks that require competence over the long term by their attribute of grit, which includes tenacity [18]. As defined by [19], gritier individuals are optimistic and committed to their work. When people learn to prioritize their goals and allocate their time and effort effectively, their grit improves [20].

Teachers who have “grit” can persevere in the face of obstacles and have a strong enthusiasm for their profession [21]. More precisely, teachers who exhibit grit are likely to have more successful classrooms [22, 17] and more engaged employees [23]. These are two of the effects that may be ascribed to the teachers. As was seen, tenacious educators devote a lot of time and effort to their work, take satisfaction in their instructional strategies, and accomplish all of this despite the difficulties inherent in their professions [24]. Even though research on language learners’ grit has been showing promising results over the last several years, very little attention has been paid to language teachers’ L2 grit and its related characteristics. According to [25], this shortcoming may be linked to the unavailability of specific methods for gauging persistence. Both [26] and [27] arrived at the same verdict: the idea of grit is reliant on its surroundings. In light of this, [17] developed the L2-Teacher Grit Scale to evaluate persistence in teaching a second language.

This study considers teaching style (TS) as an essential element for educators. As stated by [28], a teacher’s choices in terms of TS are illustrative of their pedagogical philosophy, as well as their views, values, and perspectives regarding the several components involved in the process of education and teaching. In its most fundamental form, TS incorporates all the pedagogical activities and tactics teachers do in their respective classrooms [29]. As stated by [4], the formation of these procedures is influenced by a mix of personal and professional factors. Several categorization systems have been offered to clarify the idea of TS, with [30] being the most comprehensive and well-known. Grasha’s classification positions TS between the extremes of teacher-centered and student-centered forms of teaching and presents five unique teaching styles: (1) expert, (2) formal authority, (3) personal model, (4) facilitator, and (5) delegator.

TS between the extremes of teacher-centered and student-centered methods of teaching. The terms expert, formal authority, and personal model all fall within the category of teacher-centered TS, while the terms facilitator and delegator point to student-centered TS. Educators who see themselves as experts and plan activities for their classes around disseminating comprehensive knowledge are more likely to adopt an expert mode of instruction. On the other hand, the formal authority approach to teaching necessitates instructors to adopt authoritative roles, overseeing their students while placing less emphasis on the emotional factors that students may bring into the learning environment.

Educators who choose the personal model approach expect their students to emulate and adapt their skills and procedures. The instructional course, known as the facilitator method, emphasizes the importance of self-study, self-evaluation, and self-discovery for students. Additionally, this approach highlights the role of the educator in assigning tasks that foster learner autonomy. Educators who use a delegator teaching style strategically design classroom activities that promote student collaboration, cultivating their sense of accomplishment [29]. In this regard, [30] proposed that introverted instructors are more likely to give their students independent work and written assignments, while extroverted instructors are more likely to prioritize group activities and spoken instruction in their courses. In addition, [31] discovered that TS is affected by the gender of instructors, their teaching experience, and the dominant side of their brain.

Work engagement, defined as the degree to which an employee feels emotionally invested in and derives satisfaction from their work [32], is an affective-motivational concept. Simply said, employee engagement occurs when someone commits time, energy, and enthusiasm to their job [33]. This affective-motivational construct, work engagement, prioritizes enthusiasm and participation [34]. It’s been linked to issues like dedication to one’s work, agency in one’s position, and job satisfaction [35]. In the workplace, [36] introduced the notion of “work engagement,“ which is being psychologically, mentally, and physically absorbed in one’s work.

Inaccuracies in measuring work engagement arose from researchers’ use of varied conceptualizations and definitions of the term. The present investigation used the Engaged Teacher Scale (ETS), created and validated by [37]. ETS is a multi-factor instrument for gauging how invested an individual is in their job as a teacher. This scale measures four dimensions of participation: cognitive-physical, emotional, social-among-students, and social-among-workers. The degrees of cognitive-physical engagement teachers have provided insight into the amount of mental and physical effort they put into their profession. When teachers respond positively to their work, they are said to be emotionally involved in their profession. This unique approach incorporates a measure of teachers’ social engagement (time and effort spent cultivating relationships) and the factors that contribute to it. The role of emotion control and psychological well-being as predictors of job engagement was investigated in a cross-contextual study [38]. The researchers concluded that emotion management and psychological well-being are the factors that ultimately contribute to work engagement. Nevertheless, a teacher’s psychological wellness was a better indicator of job involvement than one’s ability to regulate their emotions. In addition, it was shown that there is a connection between psychological well-being and engagement at work.

According to [39], the self-determination theory (SDT) may be seen as the theoretical underpinning of WE. Engaged individuals are motivated to bring enhanced appearance, dedication, and resourcefulness to their work, according to the SDT’s underlying concept [40]. Research conducted by [41] investigated the relationships between teacher well-being and burnout due to teaching experiences. Their investigation leads them to believe that WE and burnout are correlated negatively. Additionally, they concluded that the amount of involvement teachers showed increased as they gained more experience in the classroom.

Similarly, [42] researched China to investigate the links between WE, persistence of effort, growth mindset, and well-being. The results of their research showed that participants’ levels of well-being were significantly impacted by factors such as growth mentality, persistence of effort, growth mindset, and well-being. In addition, [38] showed that ER and psychological well-being could accurately predict teacher WE. Likewise, [43] found that an educator’s degree of dedication and self-efficacy might be utilized to predict their own and students’ well-being.

## Objectives of the current study

In dynamic and demanding educational environments, educators in general, and specifically university professors, encounter a range of emotional encounters inside their professional settings. During such instances, individuals need self-aid frameworks to facilitate their ability to make informed decisions and take appropriate actions. From the perspective of expectancy-value theory and self-efficacy, it is anticipated that university educators would maintain their motivation and exert more effort to achieve the predetermined objectives, specifically concerning second language perseverance.

**Fig. 1:**
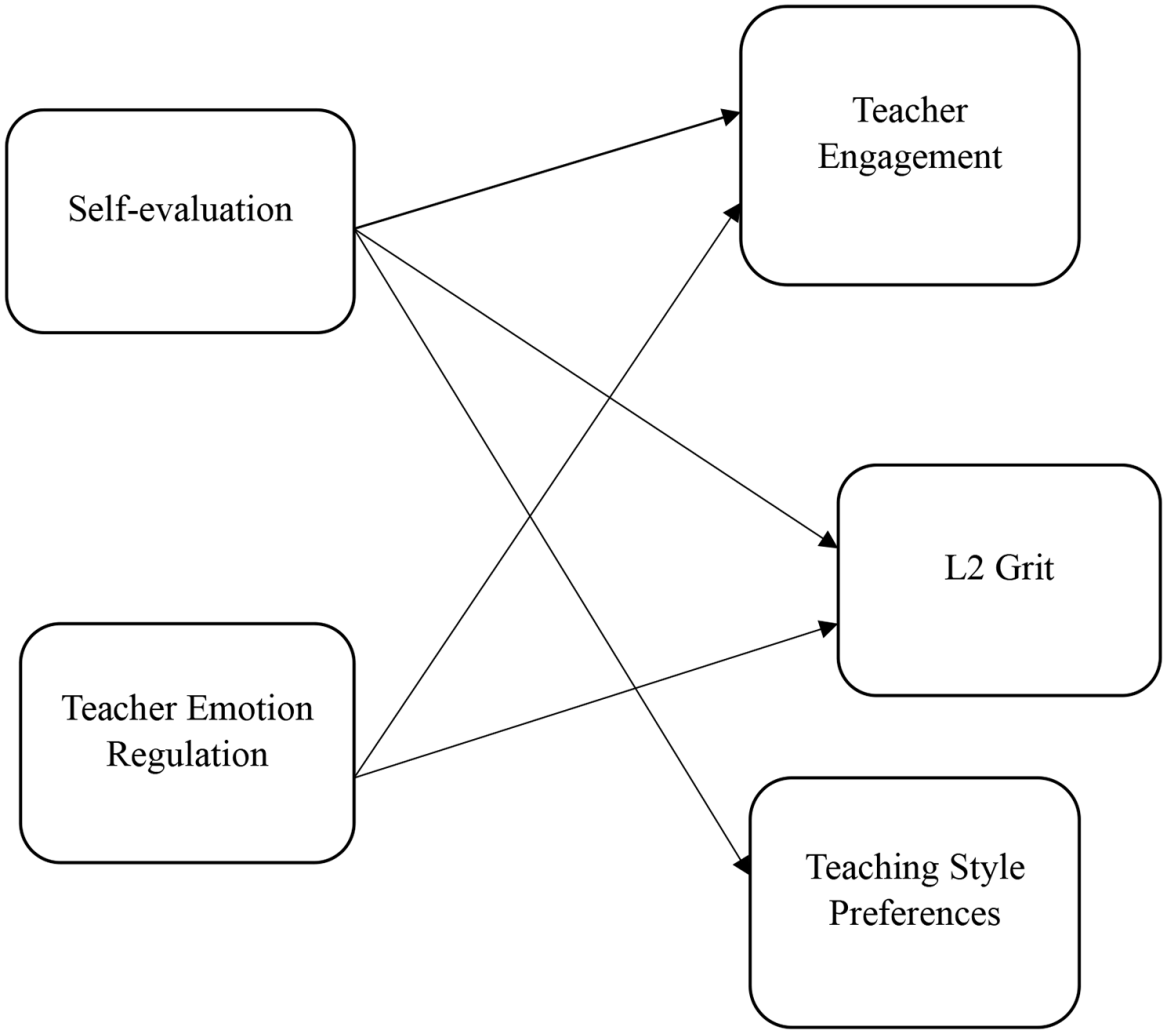
The suggested model

However, the relationship between university TER and S-E is an area that has not been well studied and requires greater investigation. The above statement holds for L2TG, particularly highlighting its emergence in 2021 and underscoring the pressing need for more scholarly research in the EFL setting. This study sought to investigate the contributions of TER, S-E, L2TG, TS, and WE in the Chinese EFL setting because of the importance of these dimensions in bolstering language teaching and the lack of studies studying their interrelationships. A conceptual model depicting the dynamic interaction of TER, S-E, L2TG, TS, and WE was built by drawing on relevant literature and theoretical frameworks. Figure 1 illustrates the connections between TER, S-E, L2TG, TS, and WE to make the ideas mentioned earlier more evident. Confirmatory Factor Analysis (CFA) and Structural Equation Modeling (SEM), two robust statistical methods often used to investigate the structural validity of latent variables and correlations among multiple variables, were then applied to the suggested model. The objectives of this study informed the formulation of the following research inquiries (RIs):

RI 1: Does teacher self-evaluation offer an understanding into teacher engagement at work, L2TG and teaching style preferences for EFL teachers?

RI 2: Does teacher emotion regulation offer an understanding into teacher engagement at work, L2TG, and teaching style preferences for EFL teachers?

## Methodology

### Participants

This research surveyed 399 EFL teachers working in various Chinese educational institutions (male = 291, female = 108). The group of instructors (ranging in age from 25 to 52) who volunteered to take part in the research were given a questionnaire that measured their perceived instructor TER, S-E on L2TG, TS preferences, and WE. All individuals employed by the institutions in academic positions possess either a Master’s or Bachelor’s degree or are actively pursuing a Doctorate. They specialized in several subfields within the discipline of English, including English Teaching, English Literature, and English Translation. In the initial portion of the questionnaire, the researchers of this study made it very clear that participants were under no obligation to answer any of the questions and that the confidentiality of their details would be maintained at all times.

### Instruments

The Language Teacher Emotion Regulation Inventory (LTERI) was designed and verified by [44] precisely to measure ER in language teachers. There are a total of 27 items that make up the LTERI, each of which can be answered with a score between 1 (indicating “never”) and 5 (indicating “always”). The items are grouped into six categories: situation selection, situation modification, attention deployment, reappraisal, suppression, and seeking social assistance. The Cronbach’s alpha test results that were within the acceptable range in the present analysis ranged from 0.738 to 0.885, respectively (Table 1).

The Core of Self-evaluation Questionnaire (CSEQ) was created and confirmed by [45]. This questionnaire was used to investigate the fundamental self-assessments of teachers. The total number of questions is 12, each graded based on a Likert scale ranging from 1 (strongly disagree) to 5 (strongly agree). The teachers’ averages on this statistic varied from 12 to 60, with 12 being the lowest and 60 being the highest. On this particular exam, greater results were achieved by positively evaluating oneself, and lower scores were achieved by negatively evaluating oneself. In the current investigation, Table 1 reveals that the CSEQ has a satisfactory reliability at 0.900.

EFL instructors’ L2 grit was examined using the L2-teacher grit scale **(**L2TGS) developed and validated by [17]. The questionnaire consists of two scales, each with 14 items measured on a 5-point Likert scale, assessing teachers’ classroom tenacity and enthusiasm for their work. This assessment tool was designed specifically to measure perseverance in EFL/ESL educators. Cronbach’s alpha for L2TGS was reported as 0.814, suggesting adequate reliability (Table 1).

The Grasha’s Teaching Style Inventory (TSI) from 1996 was used to determine pedagogical approach preferences. It consisted of forty questions, each of which may be answered using a Likert-type scale with seven points. The five elements make up this inventory: expert, formal authority, personal model, facilitator, and delegator teaching style. According to the current research findings, the acceptable dependability of the TSI’s various subscales varied from 0.828 to 0.893.

The Engaged Teacher Scale (ETS) was applied to gauge the participants’ engagement at work. ETS was developed and shown reliable by [37], with responses ranging from 1 (strongly disagree) to 7 (strongly agree) over 16 different questions. The four parts of ETS are as follows: cognitive engagement, emotional engagement, social engagement with students, and social engagement with colleagues. The researchers used Cronbach’s alpha to determine the internal consistency of each subscale. Coefficients between 0.838 and 0.896 were found to be satisfactory.

**Table 1:** Reliability Results of the Questionnaires

		N	Cronbach’s Alpha
The Teacher Emotion Regulation Inventory	Situation Selection	4	0.850
	Situation Modification	5	0.871
	Attention Deployment	4	0.833
	Reappraisal	5	0.863
	Suppression	4	0.738
	Seeking Social Support	5	0.885
	total	27	0.945
The Core of Self-evaluation Questionnaire	total	12	0.900
The Engaged Teacher Scale	Cognitive Engagement	4	0.896
	Emotional Engagement	4	0.877
	Social Engagement with Students	4	0.838
	Social Engagement with Colleagues	4	0.862
	total	16	0.943
The L2-teacher Grit Scale	Perseverance in Teaching	7	0.700
	Passion and Purpose in Teaching	7	0.795
	total	14	0.814
The Grasha’s Teaching Style Inventory	Expert	8	0.828
	Formal Authority	8	0.893
	Personal Model	8	0.880
	Facilitator	8	0.828
	Delegator Teaching Style	8	0.874
	total	40	0.956

### Data collection procedures

An online survey was given to a group of EFL teachers to gauge their ER, S-E, L2TG, TS, and TE levels. Only teachers who have freely completed consent papers (digitally or on paper) participated in the investigation. The participants were instructed to complete the scales based on their feelings and impressions of the workplace. For nearly three months in 2023, this information was gathered. Finally, a total of 399 replies from the EFL teachers’ subjects were collected.

### Data analysis

The collected survey data was then put through a preliminary review to see whether the model held up under scrutiny. Then, LISREL 8.80 was subjected to a structural equation modeling study to determine the relationship between ER, S-E, L2TG, TS, and TE in teaching English.

## Results

Data analysis summaries are presented in the following section, and every aspect of the report is explained. Table 2 displays the initial stage, which consists of the presentation of descriptive data.

**Table 2:** Descriptive Statistics

	N	Minimum	Maximum	Mean	Std. Deviation
Situation Selection	399	4	20	14.123	3.840
Situation Modification	399	5	25	17.211	4.663
Attention Deployment	399	4	20	13.544	3.737
Reappraisal	399	5	25	16.363	4.850
Suppression	399	5	20	13.847	3.845
Seeking Social Support	399	5	25	16.892	4.877
Teacher Emotion Regulation (TER)	399	31	135	91.980	20.499
Self-evaluation (SE)	399	13	60	39.779	10.105
Cognitive Engagement	399	4	20	12.882	4.547
Emotional Engagement	399	4	20	13.609	3.955
Social Engagement with Students	399	4	20	13.120	4.157
Social Engagement with Colleagues	399	4	20	13.431	4.002
Teacher Engagement (TE)	399	18	80	53.043	14.449
Perseverance in Teaching	399	12	35	24.664	4.672
Passion and Purpose in Teaching	399	11	35	25.807	5.388
L2-Teacher Grit	399	27	67	50.471	7.309
Expert	399	14	39	28.880	6.031
Formal Authority	399	12	40	28.957	6.913
Personal Model	399	10	40	28.564	6.614
Facilitator	399	13	40	28.045	5.819
Delegator Teaching Style	399	13	40	28.669	6.946
Teaching Style Preferences (TSP)	399	72	195	143.115	27.527

Considering TER, seeking social support (M = 16.892, SD = 4.877) was the highest. In the second instrument, the core of self-evaluation, the mean score was 39.779, and the standard deviation equals 10.105. Furthermore, Emotional Engagement was found to have the highest mean value (13.609) and standard deviation (3.955) among the TE scale’s primary variables when broken down into its component sections, and concerning L2TG, Passion, and Purpose in Teaching received the highest mean value (M = 25.807). Formal Authority (M = 28.957, SD = 6.913) was the highest in the last scale, reflecting TS preferences among EFL teachers.

The Kolmogorov-Smirnov test was then applied so that an analysis of the data’s normal descriptions could be conducted. According to Table 3, the sig values of all instruments and their constituent parts were greater than 0.05. This held true regardless of the instrument used. As a result, one can conclude that the data followed a normal distribution, and parametric methods are therefore appropriate for data analysis.

**Table 3:** The Results of Kolmogorov-Smirnov Test

	Kolmogorov-Smirnov Z	Asymp. Sig. (2-tailed)
Situation Selection	1.315	0.063
Situation Modification	0.849	0.466
Attention Deployment	0.908	0.382
Reappraisal	1.029	0.240
Suppression	1.294	0.070
Seeking Social Support	0.612	0.848
Teacher Emotion Regulation (TER)	0.436	0.991
Self-evaluation (SE)	0.536	0.936
Cognitive Engagement	1.183	0.122
Emotional Engagement	0.843	0.476
Social Engagement with Students	1.023	0.246
Social Engagement with Colleagues	1.172	0.128
Teacher Engagement (TE)	0.704	0.705
Perseverance in Teaching	0.829	0.498
Passion and Purpose in Teaching	1.027	0.242
L2-Teacher Grit	0.902	0.391
Expert	1.125	0.159
Formal Authority	0.836	0.486
Personal Model	0.914	0.374
Facilitator	0.803	0.540
Delegator Teaching Style	0.931	0.351
Teaching Style Preferences (TSP)	0.645	0.800

Based on the information shown in Table 2, every instrument and every one of its subscales had a sig value higher than 0.05. Since the data follow a normal distribution, parametric approaches might be used to analyze them.

In this research, a Pearson product-moment correlation was used to investigate the degree to which TER, S-E, TE, L2TG, and TS are associated with one another.

**Table 4:** The Correlation Coefficients between the subscales of TER, S-E, TE, L2TG, and TS

	Situation Selection	Situation Modification	Attention Deployment	Reappraisal	Suppression	Seeking Social Support	Self-evaluation	Teacher Engagement	L2-Teacher Grit	Teaching Style Preferences
Situation Selection	1.000									
Situation Modification	0.554**	1.000								
Attention Deployment	0.623**	0.566**	1.000							
Reappraisal	0.664**	0.614**	0.603**	1.000						
Suppression	0.589**	0.589**	0.655**	0.612**	1.000					
Seeking Social Support	0.594**	0.673**	0.551**	0.544**	0.458**	1.000				
Self-evaluation	0.712**	0.688**	0.631**	0.723**	0.604**	0.593 **	1.000			
Teacher Engagement	0.886**	0.894**	0.920**	0.942**	0.821**	0.839 **	0.466**	1.000		
L2-Teacher Grit	0.723**	0.814**	0.758**	0.783**	0.684**	0.694 **	0.524**	0.603**	1.000	
Teaching Style Preferences	0.634**	0.673**	0.613**	0.591**	0.542**	0.573 **	0.490**	0.657**	0.703**	1.000

** .Correlation is significant at the 0.01 level (2-tailed)

Following the data presented in Table 4, substantial links existed between the different aspects of the subscales of LTER, S-E, TE, L2TG, and TS. That is to say, there was evidence of beneficial connections that were statistically significant between TE and situation selection (r = 0.886), situation modification (r = 0.894), attention deployment (r = 0.920), reappraisal (r = 0.942), suppression (r = 0.821), seeking social assistance (r = 0.839), as well as self-evaluation (r = 0.466). Moreover, situation selection (r = 0.723), situation modification (r = 0.814), attention deployment (r = 0.758), reappraisal (r = 0.783), suppression (r = 0.684), seeking social assistance (r = 0.694), as well as self-evaluation (r = 0.524) were found to have a significant correlation with L2TG. In addition, meaningful relationships were found between the dimensions of TER, S-E, and TS preferences. The following connections were found to exist: situation selection (r = 0.634), situation modification (r = 0.673), attention deployment (r = 0.613), reappraisal (r = 0.591), suppression (r = 0.542), seeking social assistance (r = 0.573), as well as self-evaluation (r = 0.490).

The LISREL 8.80 statistical program was utilized to conduct the analyses of CFA and SEM, which analyzed the causal relationships between S-E, TER, TE, L2TG, and TS. Several measures, such as the chi-square magnitude, the Root Mean Squared Error of Approximation (RMSEA), the Comparative matched Index (CFI), the good fit Index (GFI), and the Nominal Fit Index (NFI), were used to evaluate the model’s closeness to the data. There should not be statistical significance when using the chi-square test, and the chi-square to degrees of freedom (df) ratio should be less than 3. Values of RMSEA below 0.1 are generally seen as acceptable, according to [46]. Furthermore, it contends that a cutoff value of 0.90 or greater should be used for the NFI, GFI, and CFI.

The results, summarized in Table 5, show that all of the fit levels (Model 1) were within the acceptable limits, including the chi-square/df ratio (2.781), the root-mean-squared error of approximation (RMSEA) (0.070), the goodness-of-fit (GFI) (0.941), the nominal fit index (NFI) (0.933), and the CFI (0.927). Table 4 further shows that the chi-square/df ratio (2.961), RMSEA (0.071), GFI (0.937), NFI (0.933), and CFI (0.924) all meet the requirements for a good fit concerning Model 2.

**Table 5:** Model Fit Indices

Fitting indexes	$\varvec{\chi }2$	$\varvec{d}\varvec{f}$	$\varvec{\chi }2/\varvec{d}\varvec{f}$	RMSEA	GFI	NFI	CFI
Cut value			< 3	< 0.1	> 0.9	> 0.9	> 0.9
Model 1	990.01	343	2.886	0.069	0.941	0.944	0.956
Model 2	3243.25	1133	2.863	0.068	0.951	0.950	0.944

**Table 6:** Summary of the Findings in Model 1

Paths			Path coefficient	T Statistics	Test results
Teacher Emotion Regulation	→	Teacher Engagement	0. 87	28.33	Supported
Teacher Emotion Regulation	→	L2-Teacher Grit	0.73	20.56	Supported
Teacher Emotion Regulation	→	Teaching Style Preferences	0.59	12.40	Supported
Self-evaluation	→	Teacher Engagement	0.44	5.87	Supported
Self-evaluation	→	L2-Teacher Grit	0.51	9.75	Supported
Self-evaluation	→	Teaching Style Preferences	0.47	7.53	Supported

**Fig. 2:**
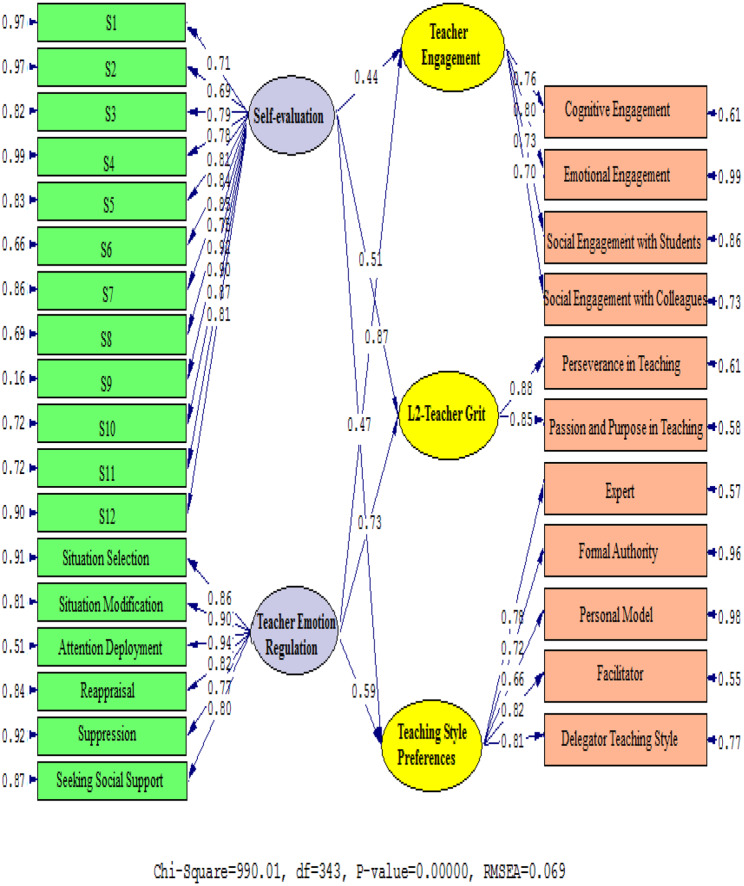
Path coefficient values expressed schematically (model 1)

**Fig. 3:**
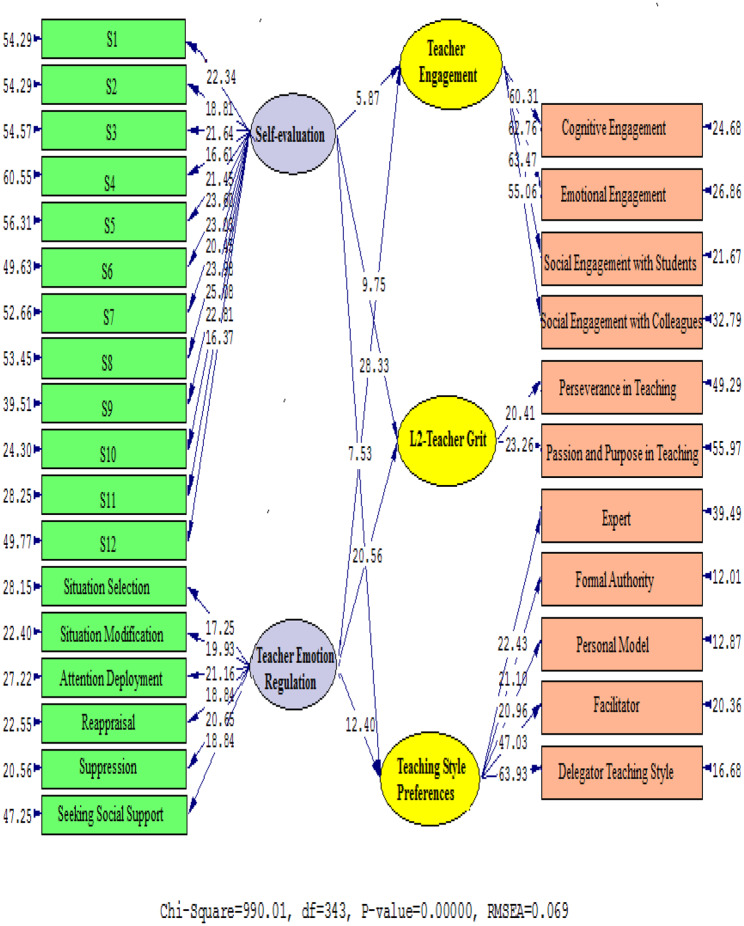
T Values for Path Coefficient Significance (Model 1)

Figures 2 and 3 (Table 6) graphically depict the relationship between the factors. Standardized estimates and t-values are presented to examine the correlation between TER and TE (β = 0. 87, t = 28.33), L2TG (β = 0. 73, t = 20.56), and TS (β = 0.59, t = 12.40). In addition, there is a positive relationship between S-E and TE (β = 0.44, t = 5.87), L2-Teacher Grit (β = 0.51, t = 9.75), and TS (β = 0.47, t = 7.53).

**Table 7:** Summary of the Findings in Model 2

Paths			Path coefficient	T Statistics	Test results
Situation Selection	→	Teacher Engagement	0. 86	26.12	Supported
Situation Modification	→	Teacher Engagement	0.88	26.62	Supported
Attention Deployment	→	Teacher Engagement	0.91	29.45	Supported
Reappraisal	→	Teacher Engagement	0.93	30.77	Supported
Suppression	→	Teacher Engagement	0.80	22.37	Supported
Seeking Social Support	→	Teacher Engagement	0.83	24.86	Supported
Situation Selection	→	L2-Teacher Grit	0.71	16.68	Supported
Situation Modification	→	L2-Teacher Grit	0.79	21.95	Supported
Attention Deployment	→	L2-Teacher Grit	0.74	19.88	Supported
Reappraisal	→	L2-Teacher Grit	0.77	21.28	Supported
Suppression	→	L2-Teacher Grit	0.66	14.31	Supported
Seeking Social Support	→	L2-Teacher Grit	0.68	14.72	Supported
Situation Selection	→	Teaching Style Preferences	0.62	13.73	Supported
Situation Modification	→	Teaching Style Preferences	0.65	14.09	Supported
Attention Deployment	→	Teaching Style Preferences	0.60	12.44	Supported
Reappraisal	→	Teaching Style Preferences	0.58	11.96	Supported
Suppression	→	Teaching Style Preferences	0.53	10.47	Supported
Seeking Social Support	→	Teaching Style Preferences	0.56	11.31	Supported
Self-evaluation	→	Teacher Engagement	0.44	5.98	Supported
Self-evaluation	→	L2-Teacher Grit	0.51	9.83	Supported
Self-evaluation	→	Teaching Style Preferences	0.47	7.65	Supported

**Fig. 4:**
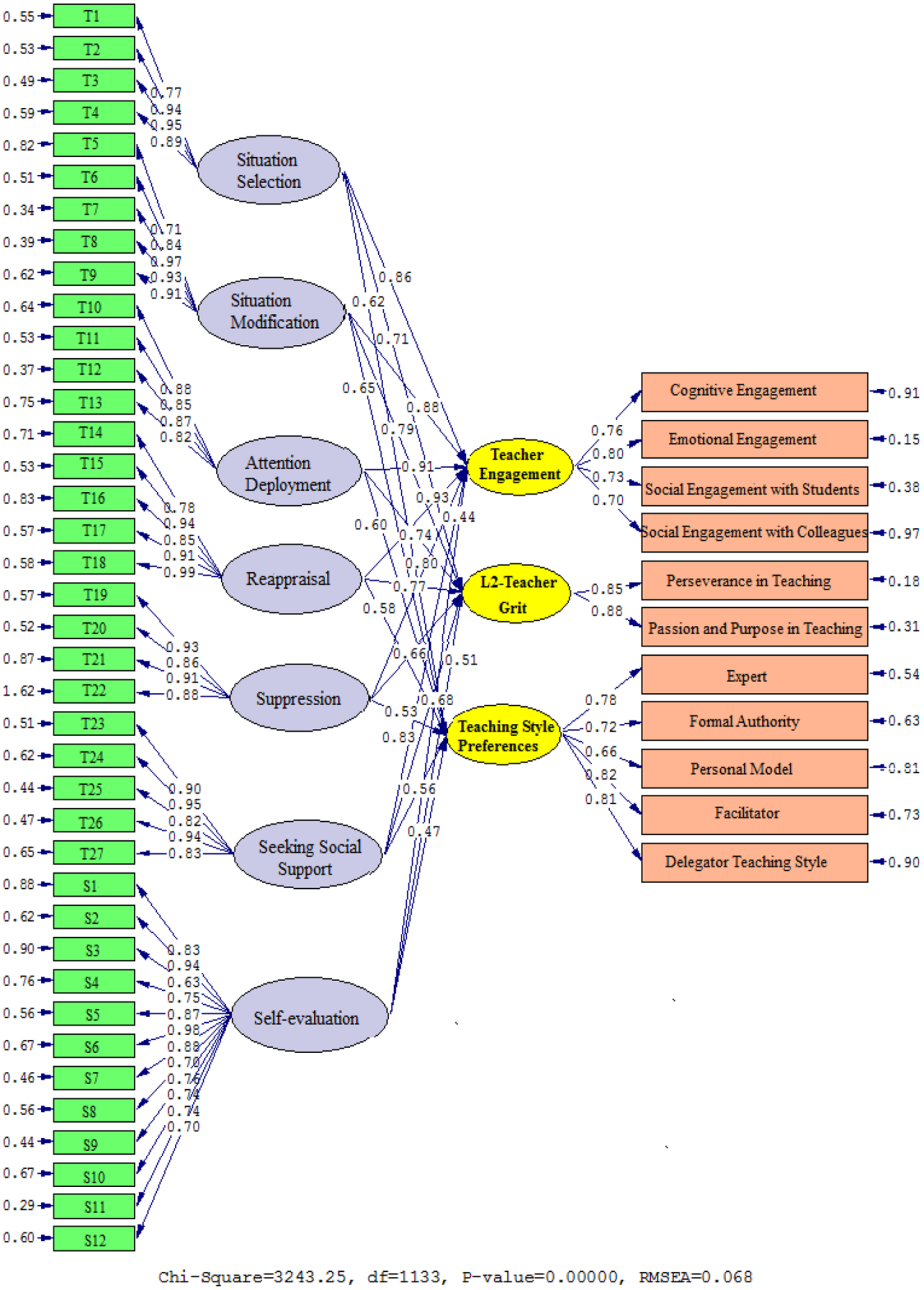
Path coefficient values expressed schematically (model 2)

**Fig. 5:**
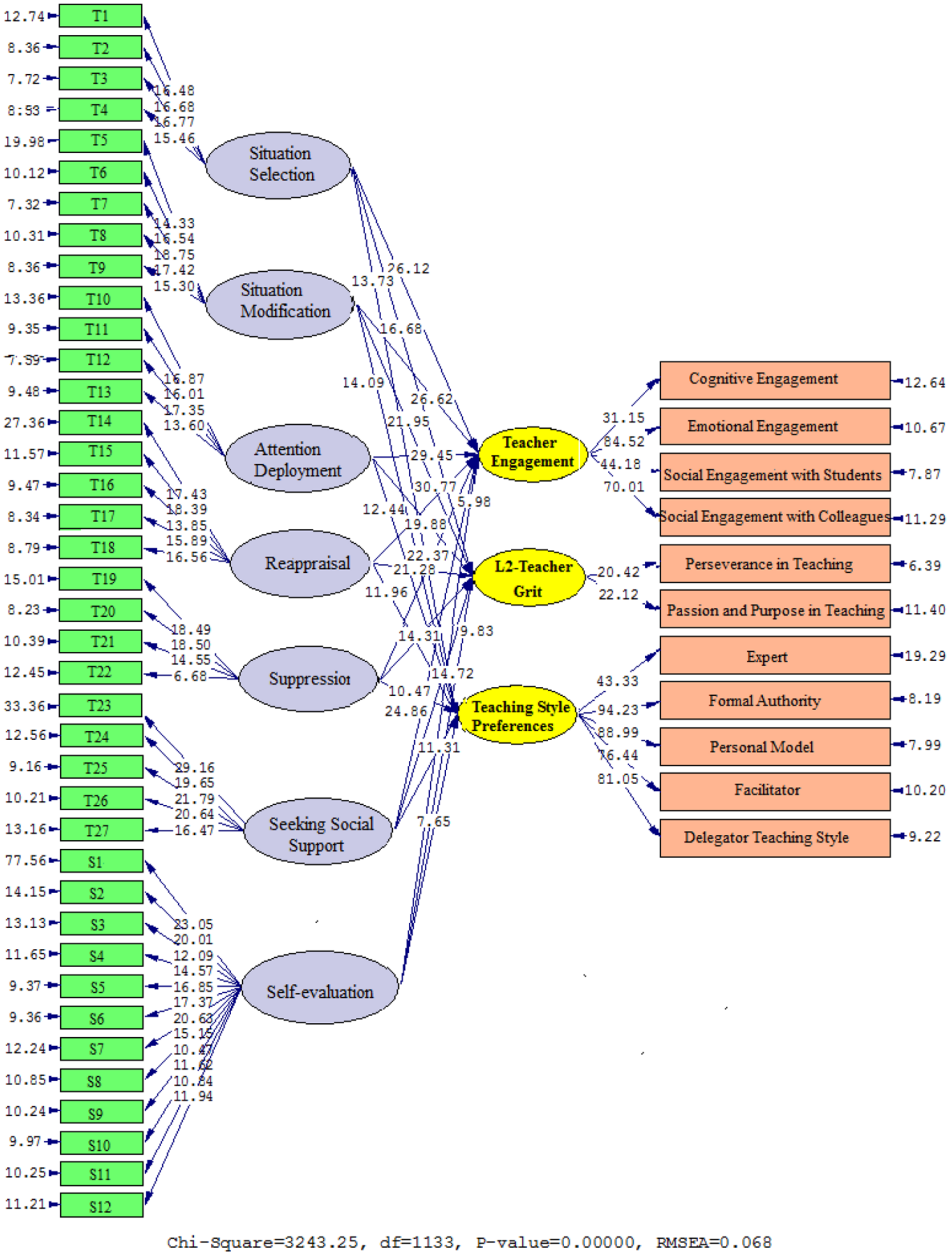
T values for path coefficient significance (model 2)

The interactions between situation selection, situation modification, attention deployment, reappraisal, suppression, seeking social assistance, S-E, TE, L2TG, and TS are graphically shown in Figs. 4 and 5 (Table 7), which show the path values of the coefficients provided by Model 2. Correlations were found between TE and situation selection (β = 0. 86, t = 26.12), situation modification (β = 0.88, t = 26.62), attention deployment (β = 0.91, t = 29.45), reappraisal (β = 0.93, t = 30.77), suppression (β = 0.80, t = 22.37), seeking social assistance (β = 0.83, t = 24.86), and self-evaluation (β = 0.44, t = 5.98). L2TG was found to be related to factors including situation selection (β = 0.71, t = 16.68), situation modification (β = 0.79, t = 21.95), attention deployment (β = 0.74, t = 19.88), reappraisal (β = 0.77, t = 21.28), suppression (β = 0.66, t = 14.31), seeking social assistance (β = 0.68, t = 14.72), and self-evaluation (β = 0.51, t = 9.83). Furthermore, there is a positive relationship between situation selection (β = 0.62, t = 13.73), situation modification (β = 0.65, t = 14.09), attention deployment (β = 0.60, t = 12.44), reappraisal (β = 0.58, t = 11.96), suppression (β = 0.53, t = 10.47), seeking social assistance (β = 0.56, t = 11.31), and self-evaluation (β = 0.47, t = 7.65).

## Discussion

This study aimed to examine the strength of the association between S-E, TER, TE, L2TG, and TS. To this end, 399 EFL teachers from China took part in this study, and the data was analyzed via CFA and SEM. The findings of this research indicated that EFL instructors and S-E, TER, TE, L2TG, and TS select a favorable and statistically significant association between the TS. To be more precise, the status of the S-E and TER might be used to predict the TE, L2TG, and TS of EFL instructors. The first question that was asked in the research was to determine whether or not the S-E of the EFL teachers could provide any insight into their TE, L2TG, and TS. The second study question was to investigate whether or not the EFL teachers’ ER provided insight into their TE, L2TG, and TS. The following is a discussion of the discoveries relating to this topic:

The results of the first study question indicate that S-E among EFL instructors has the potential to enhance their teaching effectiveness in the workplace. The participants have gained a heightened understanding of their professional challenges and uncertainties using the S-E framework. This has facilitated the cultivation of more profound cognitive processes and the adoption of more efficient problem-solving approaches. Consistent with previous scholarly investigations [47–49], the present study revealed a substantial association between instructors’ cognitive processes and evaluative judgments and their work engagement. Following the findings of [50], it was shown that there exists a strong correlation between reflective practice and the professional growth of educators. Similarly, the study by [51] found that EFL teachers saw positive associations between their self-efficacy, proficiency in higher-order thinking skills, and preferred instructional approaches.

Furthermore, the findings demonstrated that educators who engage in reflective thinking on their instructional methods carefully consider the advantages and disadvantages of their teaching strategies. Unsurprisingly, educators who engage in reflective and critical thinking are more inclined to develop and execute effective teaching strategies that promote active engagement among their pupils. Consequently, students acquiring a second language in such educational environments have a higher level of engagement and commitment to their academic pursuits. On the contrary, the findings suggest that educational settings with limited opportunities for instructors to engage in self-reflection and self-evaluation tend to exhibit a more teacher-centered approach.

Based on the findings of the second research inquiry, TER is detrimental in defining and directing TE, L2TG, and TS. This discovery might be seen as indicative of the fact that S-E interventions and assistance have contributed to improved emotional regulation among people. The potential for instructors to effectively satisfy personal and professional expectations may have been enhanced by integrating cognitive and affective processes, leading to heightened self-awareness, self-regulation, and self-evaluation [50]. Data screening also reveals that ER influences perseverance among L2 educators (Models 1 and 2). This suggests that ER affected teachers’ dedication to their profession, as measured by the second component of the pedagogical triad. The positive effects of ER on teachers’ outlooks and motivation to teach are inferred, together with the positive impact on their students’ learning. In other words, the ER serves as a campus and guides optimal performance. In academic settings, [52] results corroborated, demonstrating the symbiotic link between L2TG, emotions, and academic performance. In a similar line of inquiry, [23] found that teachers’ ER is closely tied to L2TG and self-efficacy in the Chinese EFL context.

This finding suggests that language educators may strike a healthy work-life balance by using techniques for controlling their emotions. Teachers of foreign languages report higher levels of motivation and interest in their work when they experience conditions like these. These results also suggest that a language instructor’s capacity to control her emotions may increase her level of interaction with her pupils and coworkers. As a result, instructors may increase their social commitment and professional achievements by learning to control their emotions. The underlying premises of the LTER model and SDT [53] are consistent with this finding. This result is also compatible with the few existing research on teachers’ emotions and workplace involvement [54, 47, 32], which are restricted and uncommon in teaching English as a foreign language.

The present study demonstrates that effective emotion management among EFL teachers is connected with a preference for instructional techniques emphasizing the learner. These teachers actively strove to include their students in the learning process rather than serving as the only authority in the classroom. The findings of the second model led us to conclude that there is a positive association between ER and the facilitative, delegative, personal model, expert, and formal authority teaching styles. These findings are in line with the research conducted by [55–58], which shows that the emotions of educators, both as a whole and in terms of the quality of their expressions, might provide significant obstacles or possibilities for how they educate their students. This shows that EFL teachers with a high degree of ER are more likely to foster their pupils’ intellectual and emotional development when they are teaching them in the classroom.

## Conclusion

In conclusion, the present study inferred the substantial links of S-E and TER with TE, L2TG, and TS in the Chinese EFL environment. To be more specific, the hypothesis is that teachers’ ability to S-E and regulate their emotions would lead to greater levels of TE, L2TG, and TS. The results of this research have important implications for training future language teachers, both in terms of pre-service and in-service programs. In addition, policymakers should consider these findings so that they may have a complete picture of the elements that contribute to the success and failure of teachers and educational programs.

This research has significant theoretical and practical consequences for teacher educators and EFL instructors. Some training programs and alerting EFL teachers on the significance of emotions may help teachers learn more about the situational and personality drivers of the efficacy of various tactics for S-E and TER. This kind of instruction should emphasize different tactics and demonstrate the circumstances in which each one works well. Teachers of foreign languages might benefit from additional training that encourages introspection about how their personality and outlook shape the emotional management techniques they use in the classroom. This should encourage EFL teachers to adopt more constructive methods of dealing with negative emotions, improving their efficacy. Prioritizing student-centered education within EFL pedagogical practices is essential for promoting students’ autonomy and facilitating academic success.

It is essential to have information about the situational and personality factors that determine the effectiveness of ER tactics, and teacher training programs have to consider these factors. These training programs have to emphasize practicing a wide range of tactics and demonstrating the circumstances under which each is useful or ineffective. In addition, training should highlight EFL instructors’ characteristics and preferences, which may impact the efficiency of the ER tactics they use in their classrooms. This information also encourages EFL teachers to change or adjust their already used ER tactics to more positive ones. This, in turn, is anticipated to support the university professors’ S-E practices and their grit and engagement in the classroom.

To provide more clarity, it is crucial to highlight the attainment of balance via the adjustment of emotions and the implementation of S-E in the context of EFL. This emphasis is essential for promoting a state of well-being in terms of L2 perseverance and job engagement, as well as for ensuring an instructional approach that prioritizes the needs and involvement of students. In materials creation, it is crucial to include methodologies that contain not only fundamental pedagogical knowledge but also strategies targeted at cultivating the growth of emotional resilience, S-E, perseverance, and learner-centered instructional methods.

There are caveats to this study’s methodology that should be considered when analyzing its results. This study used a quantitative method; therefore, further mixed-methods research is required to provide a complete picture of the interplay between S-E, TER, TE, L2TG, and TS. Second, this study did not investigate the potential relationships between instructors’ demographic characteristics and S-E, TER, TE, L2TG, and TS. In addition, research on the effects of S-E, TER, TE, L2TG, and TS on their students’ S-E, TER, TE, L2TG, and TS is encouraged. In the future, it is conceivable to examine the likely interaction between the S-E, TER, TE, L2TG, and TS in various educational situations.

## Data Availability

The dataset of the present study is available upon request from the corresponding author.

## Abbreviations

EFL: English as a Foreign language
ELTRI: The English Language Teacher Reflective Inventory
CSAQ: The Core of Self-evaluation Questionnaire
L2TGS: The L2-teacher Grit Scale
TSI: Grasha Teaching Style Inventory
ETS: The Engaged Teacher Scale
TER: Teacher Emotion Regulation
SDT: Self-determination Theory
S-E: Self-evaluation
TS: Teaching Style
TSP: Teaching Style Preferences
L2TG: L2 Teacher Grit
CFA: Confirmatory Factor Analysis
SEM: Structural Equation Modeling
LISREL: Linear structural relations
RMSEA: The Root Mean Squared Error of Approximation
CFI: The Comparative Matched Index
GFI: The Good Fit Index
NFI: The Nominal Fit Index
